# The ubiquitin–proteasome system is an important driver of EBV-associated nasopharyngeal carcinoma progression: a meta-analysis of transcriptomic data

**DOI:** 10.1038/s41598-025-34808-4

**Published:** 2026-02-24

**Authors:** Hana Ratnawati, Ardo Sanjaya, Aldrich Christiandy, Lawrence S. Young, Sascha Ott

**Affiliations:** 1 Department of Histology, Faculty of Medicine, Maranatha Christian University, Bandung, 40164 Indonesia; 2 Department of Anatomy, Faculty of Medicine, Maranatha Christian University, Bandung, 40164 Indonesia; 3 Maranatha Biomedical Research Laboratory, Faculty of Medicine, Maranatha Christian University, Bandung, 40164 Indonesia; 4 Undergraduate Program in Medicine, Faculty of Medicine, Maranatha Christian University, Bandung, 40164 Indonesia; 5 Biomedical Sciences Directorate, Warwick Medical School, University of Warwick, Coventry, CV4 7AL UK; 6 Bioinformatics & Digital Health Services, Research Technology Platforms, University of Warwick, Coventry, CV4 7AL UK

**Keywords:** Epstein-Barr virus infections, Nasopharyngeal carcinoma, Ubiquitin–proteasome system, Tumor microenvironment, Immune evasion, Transcriptome, Biomarkers, Cancer, Computational biology and bioinformatics, Immunology, Oncology

## Abstract

Epstein–Barr virus (EBV)-associated nasopharyngeal carcinoma (NPC) is characterized by extensive immune infiltration, yet immune evasion remains a hallmark. In this study, we aimed to leverage publicly available datasets to identify EBV–host gene interactions and re-map their expression at single-cell resolution. We conducted a meta-analysis to identify differentially expressed genes, mapped these genes to EBV–host interaction data, constructed a network, and performed pathway enrichment. Single-cell RNA sequencing datasets were used to map these genes at cellular resolution. We analysed differences in cell cycle, immune signaling, cell–cell interactions, and assessed prognostic associations of UPS signature using the GEPIA2 platform. We identified 85 EBV-interacting DEGs regulated by lytic-phase proteins. Clustering highlighted genes related to the ubiquitin–proteasome system (UPS). Single-cell analyses confirmed elevated UPS-related gene expression in NPC. UPS-High cells exhibited lower proliferative activity, enriched stemness signaling, and reduced antigen presentation and immune activation, whereas UPS-Low cells showed marked upregulation of growth-promoting pathways. High UPS expression was associated with poorer outcomes in pan-cancer, head and neck squamous cell carcinoma, and marginally significant in the small NPC datasets. The proportion of UPS-High cells varied widely across patients. UPS activity, influenced by EBV lytic proteins, is linked to immune evasion, stemness, proliferation, and adverse prognosis. These findings support UPS as a potential biomarker and therapeutic target in NPC, warranting validation and functional studies.

**Supplementary Information:**

The online version contains supplementary material available at 10.1038/s41598-025-34808-4.

## Introduction

Nasopharyngeal carcinoma (NPC) is a relatively rare but highly aggressive malignancy arising from the epithelial lining of the nasopharynx. In Southeast Asia and China, it ranks among the top ten cancers by incidence and mortality, with age-standardized incidence rates in China of 2.4 per 100,000 person-year, and in Indonesia, 6.2 per 100,000. However, the age-standardized mortality rate due to NPC in Indonesia is the highest in Southeast Asian countries, at 4.3 per 100,000, triple the mortality rate in China. An estimated 87,000 new cases and 51,000 deaths occur annually, underscoring its significant regional burden^1–3^. According to the WHO, an estimated 120,434 new NPC cases and 73,482 deaths occurred in 2022^4^. Therefore, a better understanding of the molecular mechanisms underlying NPC is critical to improving diagnostics and developing new therapeutic targets.

A defining feature of NPC in endemic regions is its consistent association with Epstein–Barr virus (EBV) infection, especially in the non-keratinizing subtype. EBV is a gamma-herpesvirus capable of latent and lytic infection in epithelial cells. Although the lytic phase typically triggers full viral replication and immune activation, it is transient in NPC and may still contribute to genomic instability and inflammation^5–7^. In contrast, latent infection predominates in NPC, characterized by restricted viral gene expression, including *EBNA1*, *LMP1*, *LMP2*, *EBERs*, and *BART* microRNAs, which modulate oncogenesis and immune evasion^2,8^.

A hallmark of EBV-associated NPC is the dense infiltration of immune cells, such as neutrophils, natural killer (NK) cells, regulatory T cells (Tregs), macrophages, dendritic cells, and B cells^9^. Although tumor-infiltrating leukocytes (TILs) are generally linked to improved outcomes in many cancers, their role in NPC is more complex. Despite the abundant immune presence, these cells often fail to mount effective anti-tumor responses, likely due to EBV-mediated immunosuppression^10^. The virus manipulates the tumor microenvironment (TME) through various immunomodulatory mechanisms. EBV induces immunosuppressive cytokines such as IL-10, TGF-β, and CCL20, which impair cytotoxic T cell activity and encourage regulatory immune subsets^11,12^. Among these latent proteins, LMP1 (Latent Membrane Protein 1) plays a pivotal role by activating key survival and immune-modulating pathways such as NF-κB, JAK/STAT, and PI3K/AKT, driving tumor progression^13,14^. LMP1 also upregulates PD-L1 expression, fostering T cell exhaustion^15^. LMP2A, another latent protein, activates the PI3K/AKT and MAPK pathways and promotes epithelial–mesenchymal transition (EMT), enhancing invasiveness^16,17^. EBV non-coding RNAs, such as *EBERs*, activate IL-6 and IL-10 production via pattern recognition receptors like RIG-I and TLR3, while also dampening type I interferon responses^18,19^. EBV-encoded *BART* microRNAs also suppress immune activation by targeting host transcripts involved in antigen processing and interferon signaling^20^. Together, these EBV-induced alterations profoundly impact the immune-dense TME of NPC, transforming the TME into an immunosuppressive niche that facilitates immune evasion, tumor survival, and metastasis. This paradox makes EBV-associated NPC a valuable model for exploring immune escape mechanisms in cancer.

Another pathway by which EBV supports oncogenesis is through manipulation of the ubiquitin–proteasome system (UPS), a key regulator of protein degradation, immune surveillance, apoptosis, and cell cycle control. LMP1 itself is regulated by the UPS, but this protein also regulates the ubiquitination of several proteins, such as TRAF6, NF-κB, and CHIP, resulting in its alteration of function^21^. The protein LMP1 is also involved in regulating many proteins, including LUBAC, LIMD1, and p62, which mediate selective autophagy and also disturb the ubiquitination process^22^. The UPS is central to antigen presentation as many viral antigens are processed and presented via MHC class I molecules^23,24^. Furthermore, EBV interferes with host tumor suppressor mechanisms via UPS modulation^23^. The lytic protein BPLF1 has deubiquitinase activity that suppresses pathways, including RIG-I and cGAS, thereby impairing interferon responses^24^. Altogether, EBV not only initiates NPC pathogenesis but also sustains tumor progression by actively reshaping immune and stromal dynamics. Its complex interactions with host immunity, signaling, and protein regulation suggest that targeting EBV-specific pathways, such as viral latency genes, immune checkpoints, or UPS interference, could offer novel therapeutic strategies.

To explore these mechanisms at scale, transcriptomic analysis provides a valuable approach. We aimed to leverage bulk RNA sequencing data integrated through a meta-analytic approach to allow robust identification of differentially expressed genes (DEGs) across multiple datasets. These DEGs were subsequently filtered to identify genes encoding proteins that interacted with EBV-expressed proteins. The identified genes were then mapped onto single-cell RNA sequencing data to investigate their expression at a cellular resolution. This approach was used to help elucidate the cell types in which these genes were most active, providing insights into the interplay between EBV infection and the TME . This strategy enabled us to gain a deeper understanding of the role of EBV in NPC pathogenesis and identify novel therapeutic candidates within the context of viral oncogenesis.

## Results

### Identification of EBV–Host interactions through Meta-Analysis

**Fig. 1:**
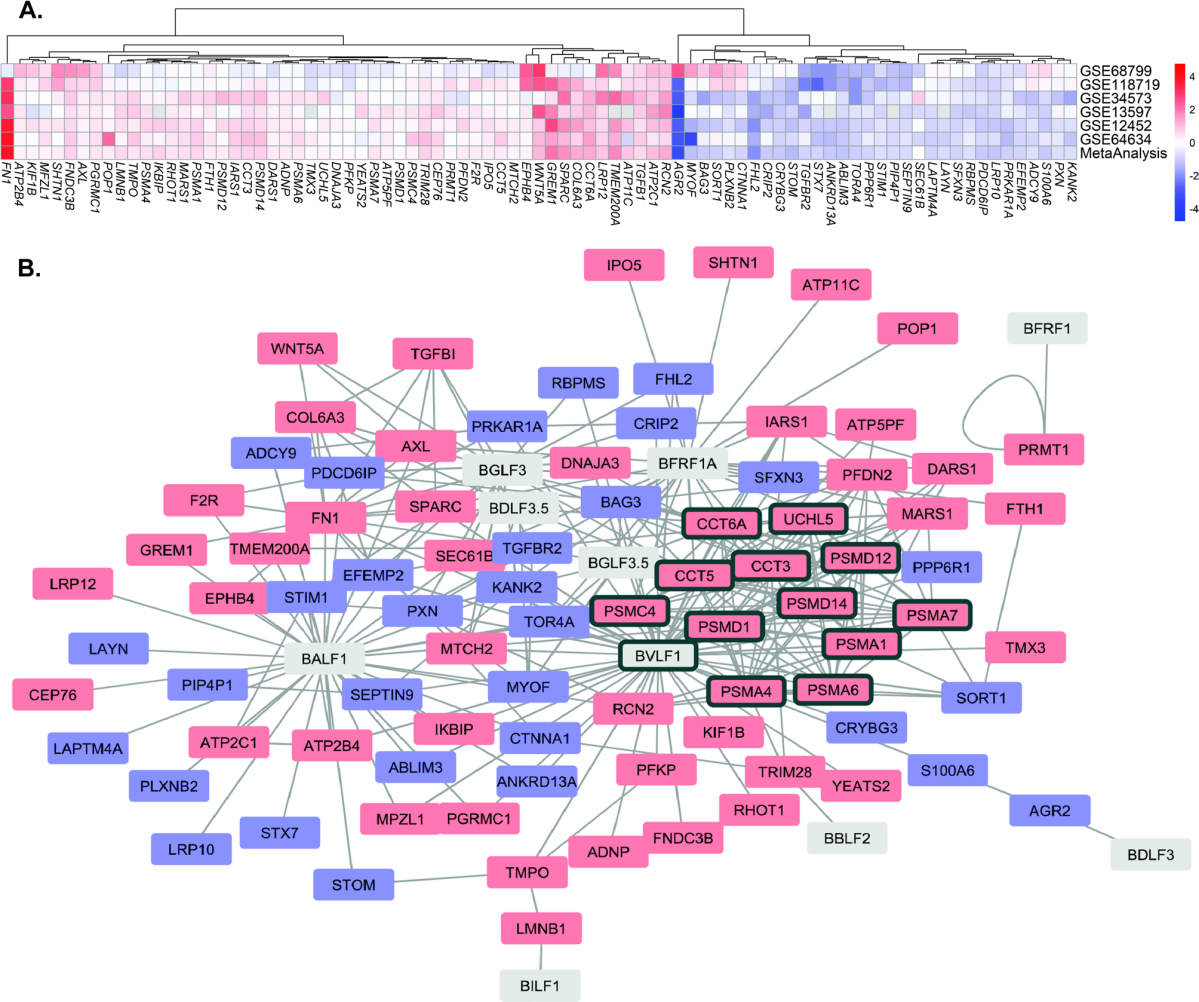
Differentially expressed genes (DEGs) and protein-protein interactions (PPI) network in nasopharyngeal carcinoma. **(A)** A heatmap displaying the logFC changes of genes interacting with the Epstein-Barr virus. The X-axis represents the individual studies and the meta-analysis results while the Y-axis represents the genes. All genes are statistically significant after the meta-analysis at the level of *p* < 0.01 after Benjamini-Hochberg correction. Note that these are data from the transcriptomic analysis and the log fold changes are small due to how the genes were averaged and the meta-analysis framework. **(B)** A PPI network demonstrating the interaction between upregulated (red) and downregulated (blue) human proteins with EBV-related proteins (grey). Key functional clusters identified by the Molecular Complex Detection (MCODE) algorithm are outlined in black boxes, highlighting central hub genes relevant to NPC development.

A meta-analysis of six publicly available datasets was performed to identify DEGs associated with nasopharyngeal carcinoma (NPC). This analysis integrated RNA-Seq and microarray datasets with a meta-regression model to correct batch effects. A total of 4004 genes were identified following multiple hypothesis correction (*p* < 0.01; Supplementary Materials 1). Genes with known interactions with the Epstein-Barr virus (EBV) were specifically selected to refine the findings further. Protein-protein interaction data for EBV-human interactions were retrieved from the VirHostNet database, yielding 548 interactions encompassing 14 EBV and 362 human proteins. Cross-referencing this dataset with the identified DEGs resulted in the retention of 85 genes (Fig. 1A). Figure 1 illustrates the PPI network of these genes and their corresponding EBV-associated interactions. Some of these EBV proteins interacting with the DEGs are involved in the EBV lytic cycle.

### MCODE network analysis reveals a UPS-related gene cluster influenced by EBV lytic proteins

Clusterization using MCODE on the PPI network identified several closely related proteins that may serve important functions in the pathogenesis of nasopharyngeal cancers. These proteins are CCT6A, CCT3, PSMA1, PSMC4, PSMA7, PSMA6, PSMA4, PSMD1, PSMD14, PSMD12, UCHL5, and CCT5. Notably, all of these proteins were influenced by several lytic proteins, including BVLF1, BGLF3, BGLF3.5, BDLF3.5, and BFRF1A. Functional enrichment analysis of the clustered genes using the Reactome database and Gene Ontology (GO) Biological Process revealed that these genes are involved in the Antigen Presentation and the Ubiquitin-Proteasomal (UPS) Pathway (Table 2; Supplementary Materials 2). This finding establishes a link between late EBV gene expression and the regulation of this essential cellular degradation and protein turnover pathway, further highlighting its potential role in NPC tumorigenesis.

**Table 2:** Enrichment results of the MCODE clustered gene from the PPI network. The top two results from each database are shown.

Databases	Term	Adjusted *P*-Value	Odds Ratio	Genes
Reactome Pathways	Cross-presentation of Soluble Exogenous Antigens (Endosomes)	*P* < 0.001	1376.483	PSMA6; PSMD12; PSMA4; PSMA1; PSMD14; PSMC4; PSMD1; PSMA7
	Regulation of Activated PAK-2p34 by Proteasome Mediated Degradation	*P* < 0.001	1376.483	PSMA6; PSMD12; PSMA4; PSMA1; PSMD14; PSMC4; PSMD1; PSMA7
GO Biological Process	Proteasomal Protein Catabolic Process (GO:0010498)	*P* < 0.001	186.57	PSMA6; PSMD12; PSMA4; PSMA1; PSMD14; PSMC4; PSMD1; PSMA7
	Proteasome-Mediated Ubiquitin-Dependent Protein Catabolic Process (GO:0043161)	*P* < 0.001	125.54	PSMA6; PSMD12; PSMA4; PSMA1; PSMD14; PSMC4; PSMD1; PSMA7

### Single-cell RNA-seq analysis identifies higher UPS-related gene expression in NPC compared to OPC

**Fig. 2:**
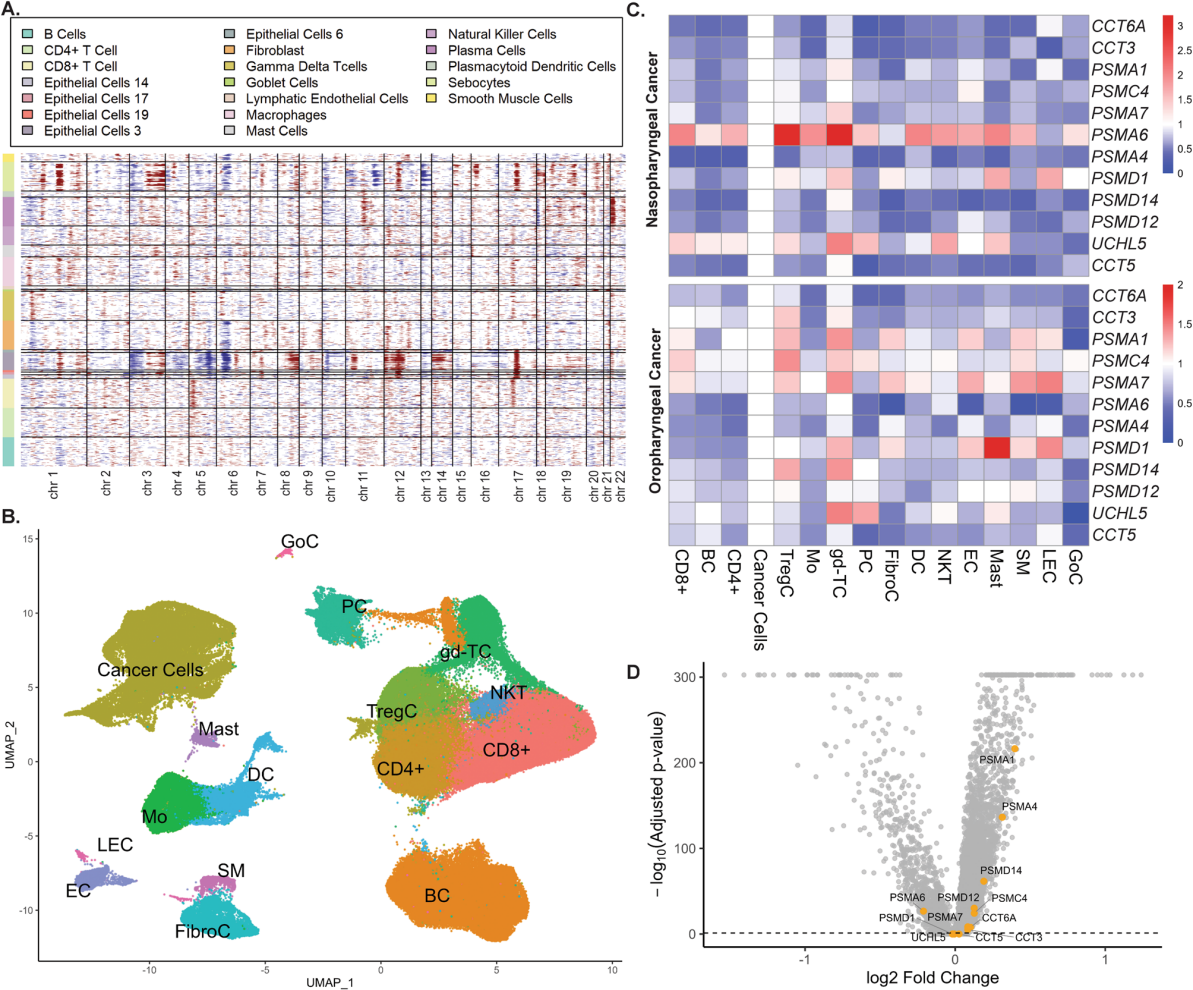
Genomic analysis and transcriptomic characterization of key genes between nasopharyngeal and oropharyngeal cancer cells. **(A)** Copy number variation (CNV) analysis of cancer cells from the combined single-cell dataset. The heatmap displays CNV alterations across several specific cell types, notably the epithelial cell clusters (14, 17, 19, 3, and 6) and the sebocyte cell cluster. Cells with gross copy number alterations were subsequently classified as cancer cells. Note that we use three cell clusters as the normal reference (endothelial cells, dendritic cells, and regulatory T-cells), hence they are not shown in the plot. **(B)** UMAP plot of single-cell clusters after relabeling. Each color represents a distinct cell type. **(C)** Heatmap of ubiquitin–proteasome system (UPS)-related genes identified from MCODE network analysis. The heatmap compares the expression of UPS-related genes across major cell clusters in nasopharyngeal carcinoma (NPC, top) and oropharyngeal carcinoma (OPC, bottom). Expression values are normalized to the cancer cell cluster, and all other clusters are shown as relative expression levels compared with cancer cells. Red indicates higher and blue indicates lower expression relative to the cancer cell baseline. Note that UPS-related genes are more highly expressed in cancer cells relative to others including CD8⁺ T cells, regulatory T cells (Tregs), and γδ T cells. **(D)** A volcano plot comparing the expression of the UPS-related genes between cancer cells from NPC and OPC. UPS-related genes are highlighted in yellow. Significant genes are those that lie above the dashed significance threshold line. Note that, except for CCT5, UCHL5, PSMD1, and PSMA6, all are significantly upregulated in NPC. Cell Type Abbreviations: BC: B-cells; DC: Dendritic cells; CD4+: CD4 + T-cells; CD8+: CD8 + T-cells; EC: Endothelial cells; FibroC: Fibroblasts; gd-TC: Gamma Delta T-cells; LEC: Lymphatic endothelial cells; Mast: Mast cells; Mo: Macrophages; NKT: Natural Killer T-cells; PC: Plasma cells; SM: Smooth muscle cells, GoC: Goblet Cells.

To further explore the role of the UPS-related genes in NPC tumorigenesis, we analyzed several publicly available scRNA-Seq datasets from the GEO database. Clustering was done based on the conserved marker genes (Supplementary Materials 3), which we enriched using several cell-type databases. We used the HPV-negative oropharyngeal cancer dataset as a comparator due to its close anatomical proximity and similar normal histological characteristics to nasopharyngeal carcinoma. However, the HPV-negative cancer dataset was used solely as a reference for cancer cells in this region rather than implying histological equivalence between the malignancies. Initial copy number variations (CNV) analysis identified gross copy number abnormalities in the clusters initially identified as epithelial cells and sebocytes. These CNV alterations were observed on several chromosomes, reflecting the deranged cell division and proliferation typically found in cancer cells (Fig. 2A). These alterations were interpreted as indicators of genomic instability, confirming that these clusters represented cancer cells, making them the focus of subsequent analysis. We then combined all epithelial clusters and the sebocytes into one cluster called cancer cells (Fig. 2B), thus reducing the initial number of clusters identified from 22 to 16 clusters. We then used heatmaps to visualize the expression of UPS-related genes from cancer cells to the other clusters and found that UPS-related gene expression is highly expressed compared to the rest of the cells in the tumor microenvironment (Fig. 2C). However, it is unclear whether these findings are unique to NPC. We then compared the genes clustered by the MCODE algorithm between NPC and OPC cancer cells and found statistically significantly higher expression of UPS-related genes in NPC cancer cells compared to OPC for almost all of the UPS-related genes (Fig. 2D). Note that these comparisons are derived from integrating the dataset using the Harmony algorithm. Harmony integration reduces dataset-specific variation, thereby yielding conservative fold-change estimates. In addition, averaging UPS expression across cells and patients further reduces fold-change magnitude. Despite their small fold-change, multiple proteasomal genes were differentially regulated, indicating pathway-level changes. This finding suggested NPC might have utilized the UPS system more for cancer progression than OPC. We observed that immune cell subsets, including γδ T cells, regulatory T cells, and CD8 + T cells, also exhibited elevated UPS-related gene expression. We could not exclude the possibility that EBV infection or signaling contributed to this. However, these cell types inherently relied on proteasomal activity for immune functions such as antigen processing, receptor turnover, and cytokine-mediated regulation. Therefore, their increased UPS activity might have reflected their roles in immune function, as shown by several studies^25,26^. Our results likely represented a combined effect of intrinsic immune function and possible viral influence, warranting further study.

### Intratumoral heterogeneity and clustering of NPC cancer cells

**Fig. 3:**
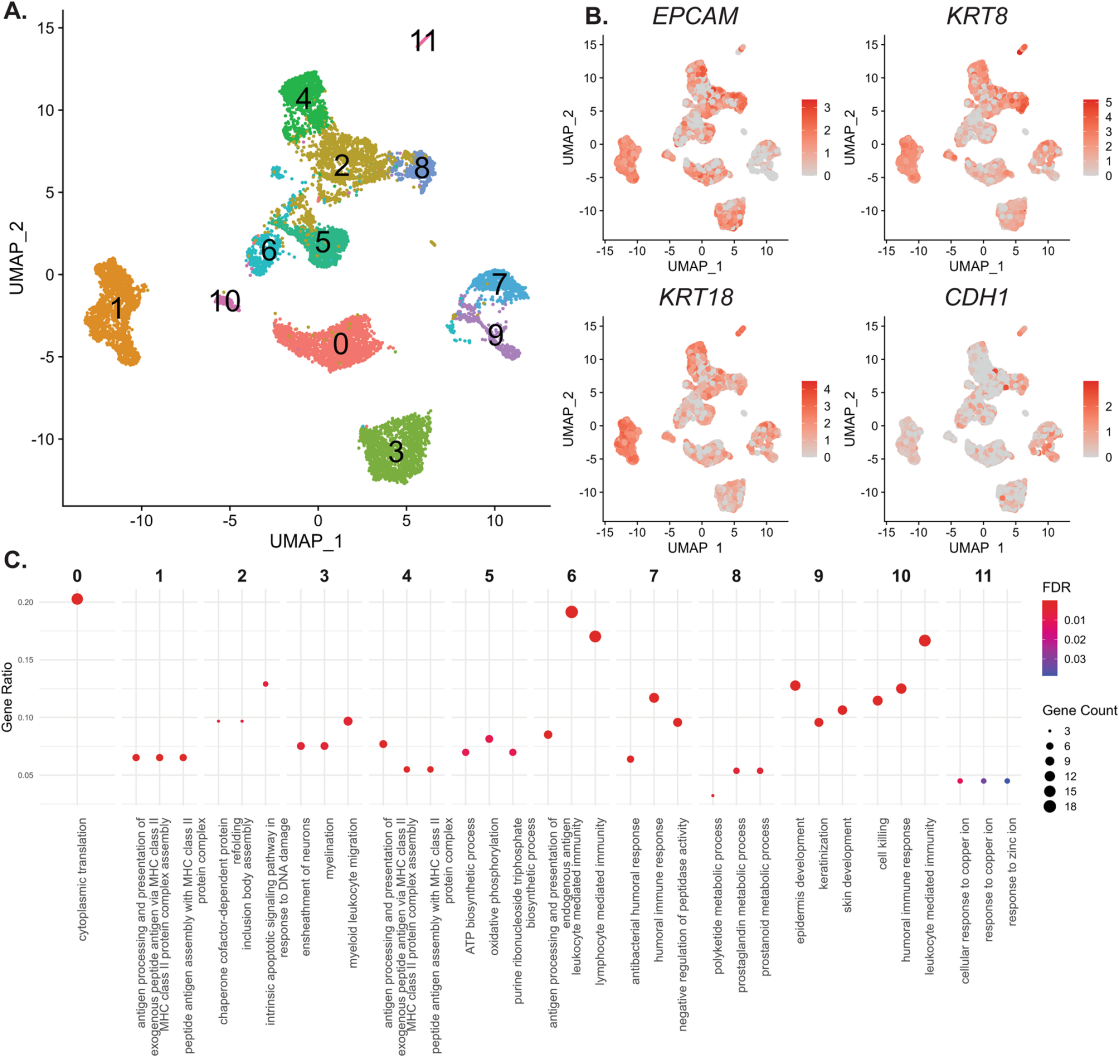
Re-clustering of NPC cancer cells and enrichment of cancer cell clusters. **(A)** UMAP plot displaying the reclustering of cancer cells from NPC. Distinct clusters are visible, highlighting the heterogeneity of NPC cancer cells. Note that from these analyses onwards, only the NPC cancer cells were analyzed. **(B)** Feature plot comparing the expression of Epithelial markers (*EPCAM*, *KRT8*, *KRT18*, and *CDH1*) across the different cancer cell clusters. Note that virtually all the cells in the tumor clusters express epithelial markers, validating their cancer identity. **(C)** Dot plots comparing the enrichment of the top marker genes for each cluster. This plot identified that each cluster is associated with distinct characteristics. For example, clusters 6 and 10 were enriched for genes associated with the immune system, while those like cluster 9 are enriched for genes associated with skin development and keratinization.

We performed a re-clustering analysis using UMAP only on the NPC cancer cells (Fig. 3A). We evaluated cluster stability across several resolution settings and visualized the resulting marker gene expression patterns using heatmaps (Supplementary Materials 4). The chosen clustering configuration produced the most distinct separation of marker expression profiles, supporting the biological validity of the identified clusters. We identified twelve different clusters, implicating significant heterogeneity in their gene expression. To validate that these cells were indeed cancer cells, we overlaid epithelial markers such as *EPCAM*, *KRT8*, *KRT18*, and *CDH1* on these clusters (Fig. 3B). The results supported the notion that these heterogeneous clusters were indeed of epithelial origin, representing cancer cells. The heterogeneity of the cancer cells was further validated by the enrichment of marker genes for each cluster (Fig. 3C). Our results showed that these cancer cells were enriched for various functions, with most of them being enriched for immune-related signaling. Clusters 1, 3, 4, 6, 7, 8, and 10 exhibited upregulation of immune-related transcripts, indicating that subsets of epithelial tumor cells might have adopted immune-interaction transcriptional programs. This suggested a potential adaptation to the immune-rich tumor microenvironment typically found in NPC.

### Correlation between UPS activity and immune signaling pathways

**Fig. 4:**
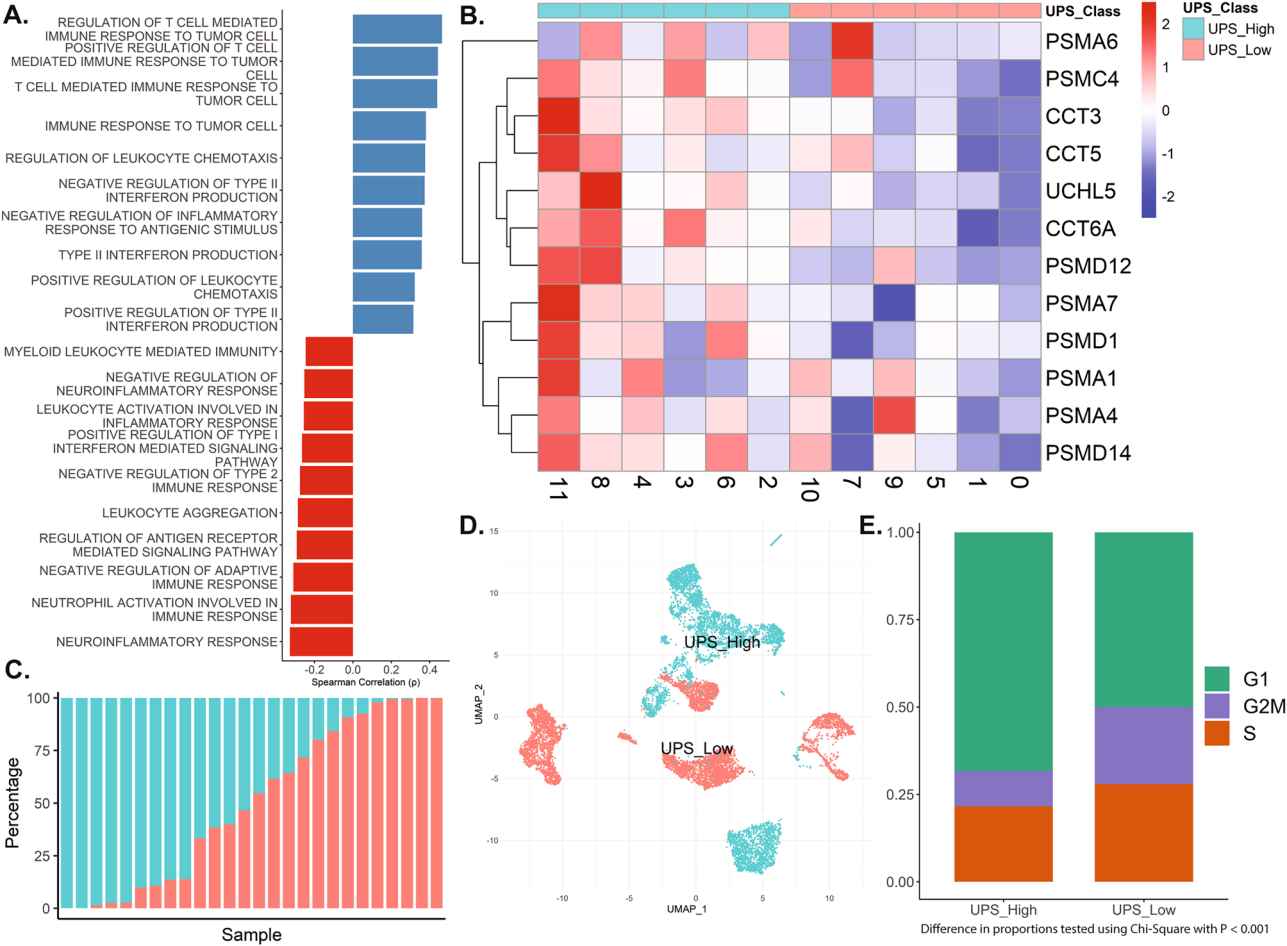
Characterization of UPS activity and its association with immune signaling, gene expression patterns, and cell cycle states. **(A)** Bar plot showing Spearman correlation coefficients between the ubiquitin-proteasome system (UPS) expression signature and AUCell-derived activity scores of immune-related GO Biological Process terms across tumor cells. Note that a higher average expression of the UPS system is associated with a higher T-cell-mediated immune response and leukocyte chemotaxis. However, the higher UPS expression is also negatively correlated with the inflammatory response, neutrophil activation, and leukocyte activation. Perhaps suggesting that UPS-high tumor cells may be more visible to immune surveillance, but also downregulate inflammatory signalling to escape immune elimination. **(B)** Heatmap comparing the expression of UPS-related genes across the different cancer cell clusters. Expression was row-scaled (z-score) to each gene’s mean expression across all clusters. Notably, clusters 2, 3, 4, 6, 8, and 11 showed increased UPS-related gene expression and are classified as UPS-High, while the remaining clusters are classified as UPS-Low. Red represents upregulated expression, while blue represents downregulated expression. **(C)** Distribution of UPS-High and UPS-Low tumor cell proportions across patient samples. Each bar represents one patient, and the segments denote the relative percentage of UPS-High (blue) and UPS-Low (red) tumor cells. Note the inter-patient heterogeneity in UPS activity. **(D)** UMAP plot showing the distribution of cancer cells after reclassification to UPS High and Low according to the expression of UPS-related genes. **(E)** Bar plot comparing the proportion of cells in different cell cycle phases between UPS-High and UPS-Low groups. A markedly higher proportion of cells in the G1 phase is observed in the UPS-High group, with a corresponding lower proportion of cells in the S phase, indicating potential differences in cell cycle regulation. Proportions were tested using a chi-square test with *p* < 0.001.

We then further validated whether the previously identified upregulated UPS system has any roles in immune signaling within NPC cancer cells. Using the AUCell package in R, we correlated the average expression of UPS-related gene expression with the pathway scores generated by AUCells (Fig. 4A). We identified a significant positive correlation between the expression of UPS-related genes and immune-related pathways, including T-cell-mediated immunity to tumor cells and the negative regulation of interferon and inflammatory responses, among others. However, note that the results of these correlation analyses are also associated with both positive and negative associations with several related immune processes. UPS-high cancer cells show positive associations with antigen-processing and proteasomal peptide-generation pathways, consistent with the well-known dependence of MHC class I processing on UPS function. However, the interferon-stimulated pathways, neutrophil activation modules, and inflammatory cytokine programs show negative correlations with UPS expression. This pattern likely reflects EBV effects, as it has been shown that the UPS-high states are associated with EBV-mediated proteasomal dysregulation^21^. The UPS system was exploited as a method to evade the immune system, potentially dampening type I interferon responses and downstream inflammatory signaling^14,27–29^. This pattern thus represents complementary aspects of EBV-associated immune dysregulation. Taken together, these results suggest that cells with high expression of UPS-related genes have a significant influence on the immune response, exhibiting both a positive association with the immune response to tumor cells and a negative response to other parts of the immune system. Further research is needed to clarify their direction.

### Classification of cancer cells into UPS-high and UPS-low subgroups

A heatmap of UPS-related gene expression (Fig. 4B) was created to compare the expression of these genes across cancer cell clusters. We then classified these clusters into UPS-High and UPS-Low based on the median of the average expression of UPS-related genes as the cutoff. Clusters 2, 3, 4, 6, 8, and 11 were classified as UPS-High, while the rest were classified as UPS-Low (Fig. 4D). UPS activity was found to be highest in Cluster 11, with similarly elevated expression in other clusters. These suggest that UPS activation is shared among several tumor subpopulations, although not equally. Note that Cluster 11 comprises a small fraction of the total cells. Nevertheless, since the classification was defined using the median UPS activity across all tumor cells, this minimizes the influence of extreme clusters on classification outcomes.

We next assessed the distribution of UPS-High and UPS-Low tumor cells across individual patient samples to determine whether these subpopulations were restricted to specific patients (Fig. 4C). Our analysis revealed that tumor cells with high or low UPS activity were distributed heterogeneously across the cohort. While some patients harbored predominantly UPS-High tumor cells, others exhibited mostly UPS-Low populations, with yet others showing a more balanced distribution between these two cell states. We then examined the distribution of cancer cells in different cell cycle phases between UPS-High and UPS-Low groups (Fig. 4E). The analysis revealed a higher proportion of cells in the S phase within the UPS-Low group, suggesting increased proliferative activity. Conversely, UPS-High cells exhibited a greater proportion in the G1 phase, indicative of a more quiescent state. These differences in proportion were tested using a chi-square test, yielding a statistically significant result (*p* < 0.001). These findings suggest that cancer cells in the UPS-High group may exhibit reduced proliferative signaling, possibly reflecting a shift toward a more quiescent, stem-like state.

### Cell–cell communication reveals distinct tumor–immune interactions

**Fig. 5:**
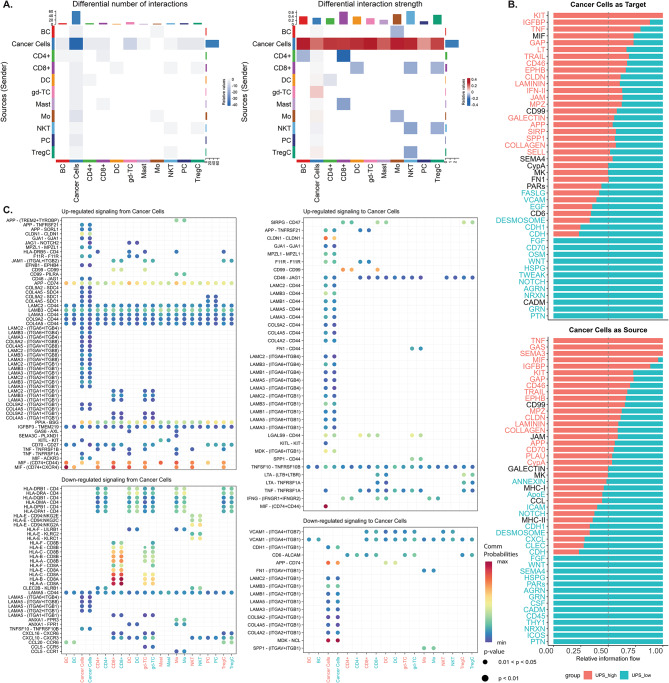
Predicted cell-to-cell interactions and differentially expressed ligand-receptor pairs between the UPS-High and UPS-Low cancer cells within the tumor microenvironment. **(A)** Heatmap illustrating the number of interactions and interaction strength between cancer and resident immune cells. Red denotes higher interactions in the UPS-High group, while blue denotes higher interactions in the UPS-Low group. Note the marked decrease in the number of self-interactions between cancer cells and the higher interaction strength between cancer cells and the resident immune cells in the UPS-High tumor cells. **(B)** Bar plots showing the relative information flow of signaling pathways where cancer cells act either as the target (top) or the source (bottom), stratified by UPS expression level. The color of each pathway label indicates statistical significance: red denotes pathways significantly enriched in UPS-High cells, while blue denotes pathways significantly enriched in UPS-Low cells. Non-significant pathways are shown in black. The relative information flow is defined as the ratio of signaling probability in one group divided by the sum of probabilities in both groups. Signaling pathways enriched in UPS-high cancer cells (red) include those related to immune evasion (MIF, TNF, GALECTIN) and cancer stemness (KIT). In contrast, UPS-low cells (blue) show higher activity in signaling pathways such as FGF, NOTCH, and WNT, implying association with cancer proliferation and growth. This shift reflects the potential for UPS-high tumor cells to adopt a more immune evasive and stem-like properties, while UPS-low cells may retain their high proliferation properties. **(C)** Bubble plot of significantly upregulated and downregulated signaling from (left) and to (right) cancer cells. The size of each circle corresponds to the p-value, while the color intensity reflects the communication probability, with warmer colors indicating stronger interactions. The x-axis denotes the cell target of the interaction of the cancer cells from the UPS-High group (red) and the UPS-Low group (blue). Note that for each upregulated or downregulated ligand-receptor pair, two columns are shown, one for UPS-High and UPS-Low. These columns represent communication probabilities in both groups. Therefore, although the ligand-receptor pairs are upregulated in one group, their predicted communication probabilities are shown in both. Notable highlights include the significant upregulation of the MIF signaling pathway in the UPS-High cancer cells compared to the UPS-Low cancer cells. Notable highlights include the upregulation and downregulation of several specific Laminin signalling pathways in the UPS-High group, suggesting the variable effect Laminin signaling may have on NPC pathogenesis. However, note that the communication probabilities of Laminin signaling are quite low in both groups. Cell Type Abbreviations: BC: B-cells; DC: Dendritic cells; CD4+: CD4 + T-cells; CD8+: CD8 + T-cells; gd-TC: Gamma Delta T-cells; Mast: Mast cells; Mo: Macrophages; NKT: Natural Killer T-cells; PC: Plasma cells; TregC: Regulatory T-cells.

To further investigate the role of UPS-related differences in cancer cell behavior, we analyzed the cancer cells from both groups and their interactions with immune cells within the tumor microenvironment. Note that these analyses represent computational prediction using CellChat. The analysis was restricted to immune cell interactions to assess differences in tumor-immune dynamics between UPS-High and UPS-Low cancer cells. Our analysis identified a marked difference in the number and strength of interactions between cancer cells and their microenvironment (Fig. 5A). The cancer cells in the UPS-High group demonstrated mostly decreased self-interactions, both with themselves and with other resident immune cells. However, the UPS-High group cells demonstrated increased communication strength compared to the UPS-Low group. Figure 5B illustrates the directional information flow of signaling to and from cancer cells, revealing enrichment of immune evasion–associated pathways (e.g., MIF, TNF, SIRP, and GALECTIN) in UPS-High cells. In contrast, UPS-Low cells exhibited significantly increased signaling in proliferation-related pathways, including WNT, FGF, and NOTCH. The statistical significance of each signaling pathway tested can be seen in Supplementary Materials 5.

Differential expression analysis of ligand–receptor pairs (Fig. 5C; Supplementary Materials 6) showed that MHC-I and MHC-II signaling pathways were relatively downregulated in UPS-High NPC cancer cells, consistent with the immune-evasive trend observed in our analysis. It is important to note, however, that both UPS-High and UPS-Low groups still express MHC-related ligand–receptor pairs, resulting in high communication probabilities in both groups. The difference therefore reflects relative expression levels, with MHC-I and MHC-II molecules expressed at lower levels in UPS-High cells, rather than a complete absence of these signaling. Another notable pathway only identified in the UPS-High cells is MIF signaling, which is responsible for the negative regulation of immune activation. The MIF signaling is shown to be highly expressed in the UPS-High group, with a high probability of signaling to all other resident immune cells, including CD4, CD8, Dendritic Cells, Macrophages, and natural killer T-cells. A notable finding is the varying regulation of laminin signaling, with the ligands being selectively upregulated while some were downregulated. These suggest the complex function of Laminin signaling with the potential to support and inhibit cancer growth.

### Elevated UPS activity predicts poorer clinical outcomes

**Fig. 6:**
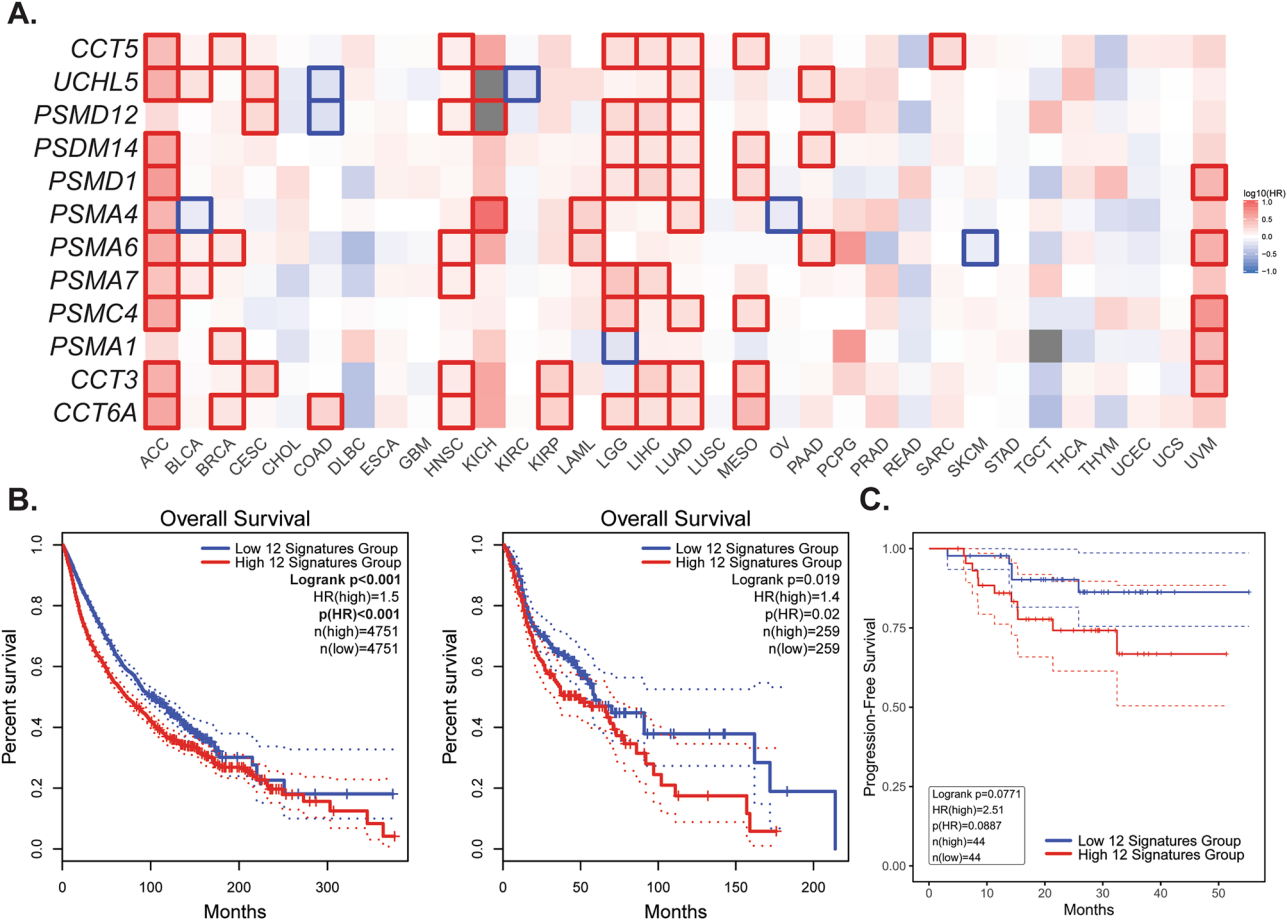
Prognostic relevance of the 12-gene UPS signature across cancers.**A.** Survival heatmap of each individual UPS gene (CCT6A, CCT3, PSMA1, PSMC4, PSMA7, PSMA6, PSMA4, PSMD1, PSMD14, PSMD12, UCHL5, and CCT5) across multiple cancer types from the TCGA pan-cancer dataset, analyzed using GEPIA2. The color scale represents the log10 hazard ratio for overall survival, with red indicating a higher risk associated with high gene expression and blue indicating a protective association. Significant associations (*p* < 0.05) are highlighted with red or blue boxes. **B.** Kaplan–Meier survival curves comparing patients with high versus low expression of the combined 12-gene UPS signature analyzed using GEPIA2. **Left**: Pan-cancer dataset (*N* = 9,502; HR = 1.5, *p* < 0.01). **Right**: Head and neck squamous cell carcinoma dataset (*N* = 518; HR = 1.4, *p* = 0.02). **C**. Kaplan-Meier survival curves of the same 12-gene UPS signatures from an NPC-specific dataset. Note that they are only marginally significant (*N* = 88; HR = 2.51; *p* = 0.0887), although these might reflect the small sample size.

Our previous analysis has shown that high UPS activity is associated with stemness features and immune evasion signaling. Therefore, we next aim to determine whether this phenotype translates into differences in patient survival. We used the GEPIA2 platform to examine the prognostic value of the 12 UPS-related genes across the available cancer types from the TCGA datasets. Each gene was individually assessed in the Survival Map module, with several showing significant associations (*p* < 0.05) with overall survival (Fig. 6A). We then analyzed the combined 12-gene signature in the full TCGA pan-cancer dataset (*N* = 9,502) and in the HNSC dataset (*N* = 518). We select this cancer as the closest available approximation to NPC. We also conducted survival analysis using an NPC transcriptomic dataset (*n* = 88) that included disease progression information^30^. In these analyses, high UPS signature expression was associated with significantly poorer survival for the pan-cancer and HNSC datasets (HR = 1.5, *p* < 0.01 for pan-cancer; HR = 1.4, *p* = 0.02 for HNSC; Fig. 6B). In the NPC dataset, the effect was marginally significant (HR = 2.51, *p* = 0.089; Fig. 6C), likely due to the limited sample size. These findings suggest that elevated UPS activity may be associated with poorer clinical outcomes across multiple cancer types, including NPC, and warrant further validation in larger datasets.

## Discussion

In this study, we investigated and identified a complex interaction between EBV infection and the ubiquitin-proteasome system in NPC. Our findings provided further evidence on EBV’s major role in modulating several carcinogenesis signaling pathways. We identified that several genes associated with the EBV lytic cycle may upregulate UPS function with a subsequent decrease in cell proliferation, downregulation of genes associated with immune response, and upregulation of genes associated with cancer stemness and immune tolerance. By integrating publicly available transcriptomics datasets, we outline the effects of EBV on NPC development and explain its potential pathogenesis.

### DEGs expressed by NPCs are influenced primarily by EBV lytic proteins

Our analysis identified the enrichment of 85 EBV-interacting genes among the DEGs found through the meta-analysis (Fig. 1A). Note that Fig. 1A represents the pooled log fold-change estimates derived from the meta-regression model. This approach integrates six transcriptomic datasets of multiple platforms and varying sample sizes and baseline expression levels. The resulting effect sizes are a conservative estimate because the model computes a weighted average log fold-change while controlling for between-study heterogeneity. However, the direction of expression change for these EBV-interacting genes was consistent. We argue that although the effect size changes are small, subtle changes can still influence biological function. Additionally, the consistent differential regulation across datasets further supports the validity of this finding in nasopharyngeal cancer.

This finding underscores the significance of viral influence in the development of nasopharyngeal cancer. Previous studies have reported that EBV induces multiple alterations to the tumor microenvironment, particularly due to its ability to infect multiple cell types^14,31^. Interestingly, contrary to previous findings that associate EBV carcinogenesis with the latent phase of the infection^32^, our findings link the lytic cycle and the associated expression of lytic proteins to EBV carcinogenesis. We found that of the DEGs identified to be EBV-related, almost all are influenced by EBV lytic proteins. Our findings align with several studies that found EBV exploits the host ubiquitination and proteasomal degradation processes to evade the immune system^33,34^. EBV also promoted the selective degradation of proteins of the immune system, such as the MHC-I and MHC-II, through its lytic proteins^34,35^ while preferentially reducing degradation of EBNA^33^. Therefore, the lytic phase of EBV infection might facilitate cancer persistence by facilitating immune escape and increasing the viral spread. It is essential to note that several recent studies have highlighted the expression of EBV lytic proteins in NPC and other virus-associated tumors, implicating these EBV proteins in the oncogenic process and immune evasion^6,36^.

The UPS is essential for regulating protein turnover and antigen processing. The dysregulation of this system is implicated in various malignancies, including those associated with viral infections such as EBV^37^. Viruses use the UPS to degrade specific cellular targets to promote their replication. However, this degradation of host proteins may have deleterious consequences for the host, such as the degradation of p53 and the retinoblastoma tumor suppressor by the human papillomavirus (HPV) infection, which predisposes these cells to cancer transformation^38^. Using HPV-negative oropharyngeal carcinoma as a control of a similar location and histology, we identified that UPS-related genes are significantly upregulated. This reflects that although cancer cells have dysregulation of UPS activity, EBV infections cause further derangement to promote their persistence and cancer progression. The importance of UPS activation in cancers was also shown by studies exploring bortezomib, a proteasomal inhibitor, in enhancing cancer cell recognition by the immune system and reducing proliferation in multiple cancers, including NPC^29,39,40^.

### Cluster analysis revealed intratumoral heterogeneity of UPS functions

Given the heterogeneity of cancer cells, we reclustered the NPC cells to examine the expression of UPS-related genes further. Initially, we identified 12 distinct cancer cell clusters, each with different gene functions enriched. We identified that the majority of cancer cells were enriched with genes regulating the immune system. These supported previous findings of dense inflammatory infiltrate and interaction in nasopharyngeal cancers^41–43^. To evaluate the influence of UPS-related genes on the immune system, we correlate the average expression of UPS-related genes with the gene enrichment score generated using AUCells. We identified that high UPS expression is positively correlated with the regulation of T-cell-mediated response to tumor cells. These findings are supported by the main function of the UPS system in antigen presentation and its importance in cancer cell recognition^25^. However, we also identified that high UPS expression is negatively correlated with markers of immune response and inflammation. These findings were confirmed by previous studies that demonstrated the UPS system as essential for regulating the immune response^42,43^. Although the results in Fig. 4A showed positive correlations with anti-tumor immunity and T-cell immunity. These correlations perhaps reflect the shared biological components of the UPS-related genes with antigen presentation and T-cell-based immunity. Further investigation using Cell-Chat in the upcoming sections examines the intricate regulation of immune signaling in nasopharyngeal cells, as UPS activation is paradoxically linked to immune evasion. In nasopharyngeal cancers, we postulate that high UPS activity, although it is associated with cancer cell recognition, also leads to immune dysregulation and evasion, which may support their growth.

Using the average expression of UPS-related genes, we classified the cancer cells into two main clusters: UPS-High and UPS-Low. Previous studies have associated proteasomal dysfunctions with cancer growth^37,40,44,45^. Consistent with previous findings, we identified statistically significant differences in the proportion of cells in the G1, G2/M, and S phases between the two groups. Higher proportions of cells were observed in the S phase and lower proportions in the G1 phase in the UPS-Low group, indicating higher proliferation. These findings suggest that cells with low UPS activity have a more rapid cell proliferation, presumably through the potential non-degradation of growth signals. Several studies have linked the deregulation of UPS activity to increased signaling activity of pathways such as Wnt^46^, NF-κB^37^, PI3K/Akt^47^, and Notch^48^. In our study, we also identified increased Wnt and FGF signaling between UPS-Low cancer cells and their surroundings (Fig. 5B). The increased activity may lead to a pro-tumorigenic environment that supports cancer growth. Although cells in the UPS-High group exhibit lower proliferative signaling, they show increased activity in immune evasion pathways and express stemness-associated markers, such as *CD44*. This phenotype suggests a subpopulation that may persist during treatment and contribute to eventual relapse. This highlights the relevance of proteasome inhibition in selectively targeting the immune-evasive, low-proliferative subpopulation. However, the heterogeneous distribution of UPS-High and UPS-Low subsets across patients (Fig. 4C) suggests that proteasome inhibition alone may not be sufficient. Effective treatment strategies may require combination therapies to eliminate both proliferative and immune-evasive tumor populations.

### Higher UPS activity is associated with immune evasion and tolerance

One of the hallmark features of cancer is the ability of cancer cells to evade immune detection^49,50^. The ability to evade the immune system is found in virus and non-virus-associated cancers^51^. However, carcinogenesis driven by viruses also adds another immune escape mechanism through viral immune evasion^51,52^. NPC was associated with marked downregulation of immune activity^34,53^. Our findings further linked these immune escape mechanisms with UPS activity, with marked downregulation of ligand-receptor pairs belonging to the MHC class I and II molecules in UPS-High cells. The downregulation of MHC class I and II signaling was observed between the cancer cells and the immune cells (CD4+, CD8+, γδ T-cells, macrophages, and regulatory T-cells), effectively making the cancer cells invisible to the immune system. Our findings are supported by previous studies identifying EBV proteins such as BDLF3^34^ and BILF1^35^, which promote the ubiquitination and degradation of MHC class I and II molecules. These observations suggest that EBV may substantially impair antigen presentation, likely through manipulation of the ubiquitin–proteasome system to enhance proteasomal degradation of immune recognition molecules.

We identified increased expression of macrophage inhibitory factor (MIF) signaling and found that this signaling pathway is highly active (and unique) in UPS-High cells. The ligand-receptor pairs *MIF-CD74 + CD44* and *MIF-CD74 + CXCR4* were found to be highly active in signaling between cancer cells and all resident immune cells, including B Cells, CD4 + T cells, CD8 + T cells, dendritic cells, γδ T cells, mast cells, macrophages, NK T cells, plasma cells, and regulatory T cells. MIF signaling has been implicated in various cancers and appears to be involved in almost all hallmarks of cancer activity^54^. A recent study by Chen et al. found that MIF may be secreted via exosomes from NPC cells, thereby promoting NPC metastasis^55^. MIF was also shown to have dual functions, with early activity eliciting an anti-tumor immune response. However, as the cancer bulk grows, it assumes a more pro-tumorigenic response, encouraging immune evasion and neovascularization^56^. A potentially interesting link between MIF and proteasomal activity may be found in research by Wang et al. on multiple myeloma (MM)^57^. The inhibition of MIF resensitizes MM cells to the use of proteasomal inhibitors. MIF has also been implicated as a key mediator of tumor immune escape, dampening T-cell responses and promoting tumor progression in multiple cancers^58^. In nasopharyngeal carcinoma, MIF has been linked with EBV infection. The increased MIF in nasopharyngeal cancer was associated with increased tumor-associated macrophage density and pro-angiogenic signalling, supporting the role of MIF as a driver of an immunosuppressive microenvironment^59,60^. These studies, combined with our findings that UPS-high NPC cells preferentially engage MIF signaling, suggest that EBV-driven UPS activation may occur in conjunction with an immunosuppressive niche and may reflect the immune escape patterns for these cells contributing to tumor persistence and disease progression. These findings highlight a potential new combination of treatment targets for nasopharyngeal carcinoma.

Interestingly, we identified Galectin-9 (*LGALS9*) as an upregulated pathway in the nasopharyngeal cancer cells, with its interaction with CD44. Galectin-9 has emerged as a key immunoregulatory molecule, recognized in previous studies as a marker and mediator of T cell exhaustion^61^. Its interaction with CD44 contributes to immune tolerance by promoting the stability and suppressive function of regulatory T cells^62^. In a study by Kam et al. (Kam et al., 2025), elevated Galectin-9 expression in NPC tumor cells was shown to confer resistance to CD8⁺ T cell-mediated cytotoxicity, highlighting its role in immune escape and poor immunotherapy response. Additionally, although not found in our data, the interaction of Galectin-9 and Tim3 has been the focus of recent research outlining its roles in immune escape and worse prognosis in nasopharyngeal cancers^63,64^. In our study, we identified that Galectin-9 signaling is upregulated in nasopharyngeal cancers in the UPS-high groups, supporting the role of this signaling in promoting immune escape.

### UPS activity is associated with both upregulation and downregulation of laminin signaling

A particular intratumoral signaling pathways were downregulated in the UPS-High cell groups, the Laminin pathways. Laminins are large αβγ heterotrimeric glycoproteins that polymerise within the basement membrane to form a scaffold linking collagen to interact with cell surfaces. Laminins are crucial in cancers because they influence cancer growth, invasion, and metastasis^65^. We identified both upregulated and downregulated laminin ligand-receptor pairs (Fig. 5C) in UPS-High NPC cells. Recent work in NPC has shown that laminin components promote proliferation, metastasis, and may contribute to an immune-suppressive tumor microenvironment. This finding links laminin signaling directly to NPC progression and immune modulation^66,67^. However, the many components of laminin signaling also represent the complex interaction between the tumor and its immediate extracellular matrix, reflecting its context-dependent role^68^, with both pro-tumorigenic effects during the loss and upregulation of laminin signaling^69^. For example, a study identified that the *LAMC2* gene is overexpressed in esophageal cancers, and its abundance correlates with nodal metastasis and poor survival^70^. However, the same study also identified that inhibition of *LAMA3* increased growth rates and migratory capacity of esophageal carcinomas, highlighting the dual roles of laminin. Multiple laminin-CD44 signaling was also observed, representing interactions between cancer cells and cancer cells with their microenvironment. CD44 is a well-established cancer stem cell marker implicated in tumor proliferation and treatment resistance through its interaction with the basement membrane and hyaluronic acid^71–73^. Furthermore, laminin signaling, particularly the expression of laminin subunits LAMB1 and laminin‑γ2, is recognized as a mediator of immune exclusion. A recent study in NPC identified that high *LAMB1* expression is associated with poor progression-free survival and correlates with reduced infiltration of CD4⁺ and CD8⁺ T cells and reduced HLA expression, suggesting an immunosuppressive niche^67^. Although not specific to NPC, a study by Li et al. identified that tumor-derived laminin‑γ2 attenuated the response to anti–PD‑1 therapies^74^. Thus, the concurrent upregulation of laminin–integrin signaling in UPS‑High cells likely contributes to a further immunosuppressive environment that reinforces resistance to therapy.

### Several classical growth factor signaling pathways are upregulated in UPS-Low cancer cells

The increased proportion of UPS-Low cancer cells in the replicative phase of the cell cycle (Fig. 4E) may be caused by upregulated growth-promoting pathways. We identified two possible growth-promoting pathways, the FGF and the Wnt pathways. UPS-Low cancer cells were shown to upregulate FGF signalling, as shown by Fig. 5B. FGF signaling derangements were shown to drive progression and resistance of cancer cells, such as lung, breast, and nasopharyngeal carcinomas^75–78^. A recent study exploring NPC identified that up to ~ 70% of tmours display high *FGF1* transcripts, correlating with tumor growth through in vitro validations^78^. FGFR3 activation also influences macrophage phenotype, further complicating its role in NPC with its high immune infiltration^79^. Another growth-promoting pathway identified is the canonical Wnt signalling. This pathway is well known in sustaining cancer-stem-cell pools and promotes cancer motility^80^ and has also been identified to play a role in nasopharyngeal carcinoma pathogenesis^81,82^. Experimental over-expression of *WNT10A* stimulates migration, invasion, and self-renewal in squamous epithelium-derived cancers and predicts poorer survival^83^. Together, these findings suggest that UPS-Low tumor cells exhibit enhanced growth-promoting signaling activity, particularly through the FGF and Wnt pathways, and likely represent the actively replicating compartment of the tumor, in contrast to the less proliferative, immune-evasive, and stem-like UPS-High population.

### High UPS activity is associated with worse prognosis in Pan-Cancer, HNSC, and NPC datasets

Our survival analysis revealed that elevated UPS gene expression is significantly associated with poorer outcomes in both the pan-cancer and HNSC datasets, and marginally significant in the small NPC dataset. These findings were supported by other recent studies that showed the Protasome system is linked with reduced overall survival and played a significant role in NPC^45,84^. A previous pan-cancer analysis has shown that expression of *PSMA1* and *PSMD11* was linked to unfavorable outcomes in more than 30% of cancer types tested^84^. Other authors have built upon these findings and created a prognostic model using UPS gene signatures in lung adenocarcinoma^85^, thyroid carcinoma^86^, and breast cancer^87^. These findings support our results, which suggest that alterations in the UPS, linked to stemness and immune evasion in NPC, are associated with adverse survival outcomes in many cancers. The inclusion of survival data from the small NPC cohort also showed that higher UPS activity is associated with a marginally significant poorer progression-free survival, supporting the proposed association between UPS dysregulation and NPC outcomes. Our findings should reinforce the UPS system as a potential biomarker and therapeutic target that deserves further experimental validation.

The association between the high UPS expression and poorer prognosis may appear paradoxical, especially since the cell groups expressing high UPS expression showed relatively lower enrichment of proliferation-related pathways compared with the UPS-low state. However, our findings suggest that UPS-high cells represent a distinct stem-like cell state with an immune-evasive phenotype. UPS-high cells are enriched for MIF signaling, GAL9–CD44 interaction, and laminin-associated extracellular matrix programs. These allow the cells to evade the immune system and potentially resist treatments that are based on cell division. This contrasts with the UPS-low cells, which display higher growth signaling, such as WNT/FGF and cell cycle signatures suggestive of a more proliferative state. The findings of poorer patient outcomes might reflect the potential of UPS-high, stem-like, tumor subpopulations that can persist after treatment and contribute to relapse and disease progression^88,89^.

**Fig. 7:**
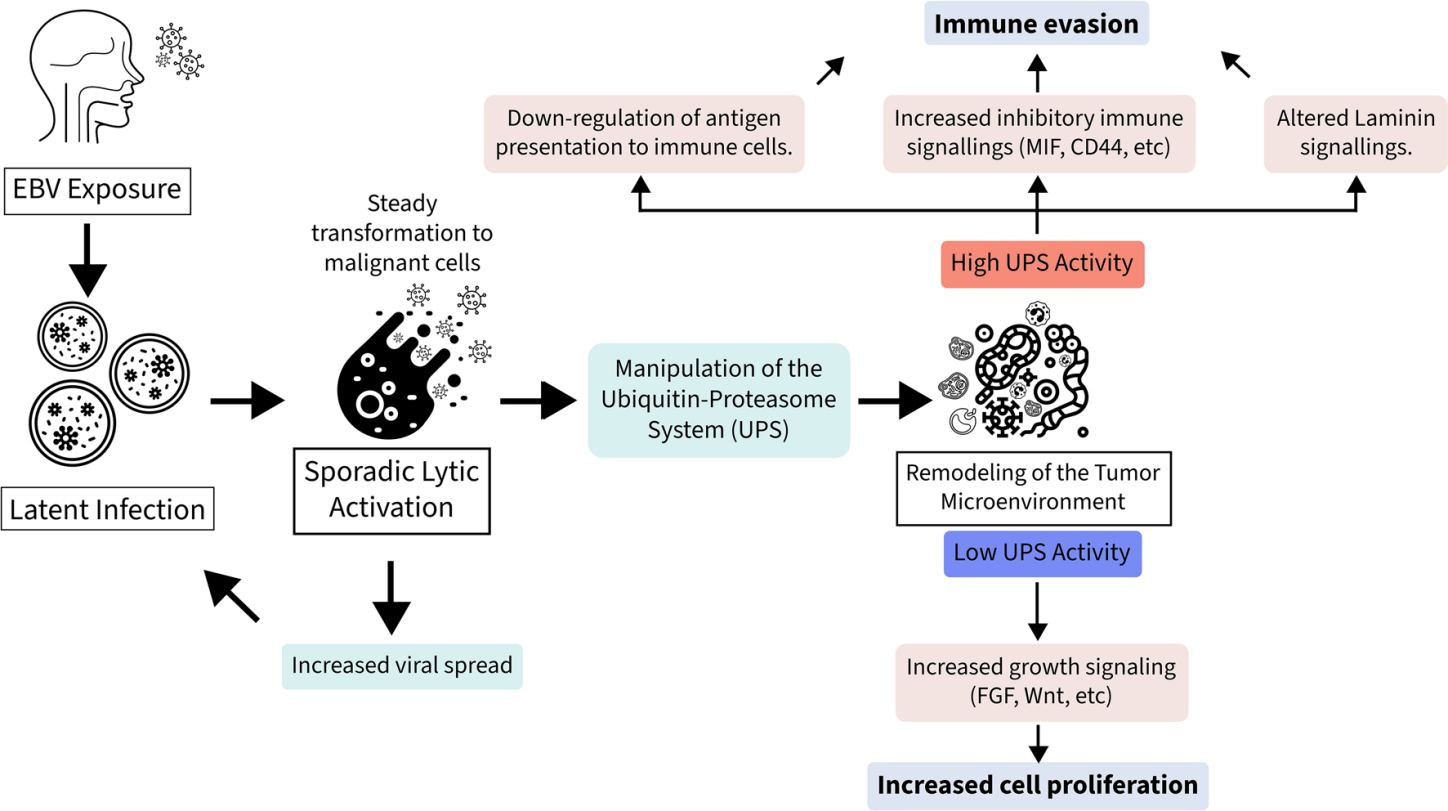
Proposed Framework of EBV-Driven NPC Progression Upon EBV exposure, latent infection is established in epithelial cells, promoting malignant transformation. Sporadic lytic reactivation enhances viral spread and may contribute to additional oncogenic events. A central mechanism identified in our study is the manipulation of the ubiquitin–proteasome system (UPS), which drives remodeling of the tumor microenvironment. High UPS activity is associated with immune evasion, characterized by downregulation of antigen presentation and increased inhibitory signaling (e.g., MIF, CD44), along with altered laminin signaling. In contrast, low UPS activity is linked to increased tumor proliferation, supported by activation of growth-promoting pathways such as FGF and Wnt.

Our findings suggest a framework in which EBV drives NPC carcinogenesis (Fig. 7). Although the traditional view emphasizes the latent EBV genes in NPC, our study identified a substantial influence of lytic phase proteins in tumorigenesis, with almost all DEGs regulated by the lytic proteins. We believe that while latent infections allow for long-term persistence of the virus, sporadic lytic activation is also essential for oncogenic transformation and immune evasion.

One of the key cellular transformations caused by EBV is the manipulation of the UPS system^45^. The dysfunctional UPS system promoted the selective stabilization of oncogenic factors, while also contributing to defective immune signaling and antigen presentation. Exploration of scRNA-seq data revealed a marked downregulation in MHC-I and MHC-II signaling, accompanied by the upregulation of other signaling pathways that promote immune escape, such as MIF. The UPS-high cells also upregulate and downregulate specific laminin signaling, highlighting their context-dependent functions. Several growth-promoting pathways, including FGF and Wnt, were upregulated in the UPS-Low group, representing the highly proliferative tumor subpopulation. We have also demonstrated that the UPS function affects the survival of pan-cancers, as well as general head and neck cancers, and even NPC, although the effect is only marginally significant. Taken together, the UPS system may contribute to the differential regulation of NPC cancer cells, leading to increased tumor persistence, proliferation, immune evasion, resistance to therapy, and adverse clinical outcomes.

However, we were unable to validate the association between EBV transcripts and UPS regulation at single-cell resolution due to the low read counts of EBV transcripts in the single-cell RNA-seq datasets. Nevertheless, our results still support the influence of EBV in tumor progression, although the exact viral program driving this remains unknown. Both proteins associated with the lytic and latent cycle of EBV have been implicated in UPS activity. Lytic proteins such as BDLF3, BNLF2a, and BPLF1 were shown to strongly influence the UPS system^24,34,90,91^. These findings suggest that lytic signaling could contribute to the UPS-high phenotype. However, EBV latent proteins may also modulate UPS activity. Type II latent proteins, such as EBNA1, LMP1, and LMP2, as well as several BART miRNAs, are known to regulate protein turnover, NF-κB signaling, and antigen presentation^92–96^. These findings suggest that UPS modulation in NPC may arise from a combination of viral and host interactions during the latent cycle, as well as from lytic reactivation. Therefore, our data suggest that UPS activation could be influenced by EBV-associated functions. However, the contributions of latent versus lytic factors in UPS activation cannot be determined. Future studies incorporating viral transcript–enriched scRNA-seq, viral protein profiling, or targeted perturbation of individual EBV genes are required to determine how EBV gene products regulate UPS activity in NPC.

We also acknowledge that the origin of proteasome upregulation cannot be attributed solely to viral activity. It is plausible that host transcription factors also contribute to the activation of the UPS system in NPC cells. Several stress-responsive regulators have been reported to induce the expression of proteasome genes. For example, NRF1 and NRF2 upregulate the proteasome system in response to proteotoxic and oxidative stress, a common condition in the tumor microenvironment^97,98^. Additionally, other oncogenic transcription factors commonly found in NPC, such as STAT3, MYC, and HIF1A, have been associated with the ubiquitin ligases and protein turnover, which is a function of the UPS system^99–101^. As our study did not incorporate a dedicated Transcription Factor regulon analysis, future work is required to determine the contribution of viral influence and host-derived factors in shaping the UPS system activity.

This study provides valuable insights into the role of the ubiquitin–proteasome system in EBV-associated nasopharyngeal carcinoma; however, several limitations should be acknowledged. First, our conclusions are based solely on transcriptomic data, including bulk and single-cell RNA sequencing, without validation at the protein level. Therefore, our key findings, such as UPS activity, antigen presentation deficits, and ligand–receptor interactions (e.g., MIF, laminin, WNT, and FGF pathways), remain inferential. Although our analysis links EBV gene expression with the upregulation of UPS-related transcripts, we recognize that this relationship is correlative and does not provide evidence of a direct interaction. Several alternative explanations include proteostatic stress or immune modulation, in addition to direct ubiquitin activation by viral proteins. However, our results are supported by a recent review showing the ability of EBV infection to manipulate the UPS for EBV-associated oncogenesis^45^. Future studies will be required to determine whether specific EBV lytic proteins directly influence UPS function in nasopharyngeal carcinoma.

Second, cell–cell communication analysis using CellChat relies on probabilistic modeling of ligand–receptor co-expression and does not confirm actual physical or functional signaling, necessitating future validation through perturbation experiments. Third, the classification of UPS-High and UPS-Low tumor cells was based on relative (median) thresholds, which may not fully reflect real-world conditions. Fourth, although our survival analysis revealed that high UPS gene expression is associated with poorer outcomes in both pan-cancer and HNSC datasets, and showed a marginally significant trend in the available NPC dataset, the limited sample size necessitates cautious interpretation of these results. Another limitation of our survival analysis is that the bulk RNA-seq analysis datasets we used lack information on tumor purity. Deconvolution tools could potentially infer approximate purity scores. However, these would introduce further errors and dataset-dependent variability. Nevertheless, the consistent findings from multiple datasets lend credibility to our findings. Incorporating deconvolution-based purity correction in future analyses may improve statistical precision and HR estimates. Lastly, although we observed substantial inter-patient heterogeneity, the number of available single-cell datasets remains limited, and the lack of integrated proteomics or clinical outcome data restricts generalizability. Future studies should include EBV infection and knockdown models to confirm viral regulation of UPS components, as well as cell-cycle and co-culture assays to evaluate the functional behavior of UPS-High and UPS-Low tumor populations. In addition, proteomic profiling, functional assays, and in vivo models will be crucial for validating the therapeutic potential of targeting UPS activity and its associated signaling pathways in NPC.

## Conclusion

This study highlights a framework of EBV-driven NPC progression, in which lytic-phase activation disrupts the ubiquitin–proteasome system (UPS), leading to altered immune signaling, reduced antigen presentation, increased stemness features, and context-dependent changes in pathways such as MIF, laminin, WNT, and FGF. These alterations appear to shape two distinct tumor cell phenotypes: UPS-High cells, which are less proliferative but more immune evasive, and UPS-Low cells, which are highly proliferative and enriched for growth-promoting signaling. Survival analysis further suggested that high UPS gene expression is associated with poorer outcomes in pan-cancer, HNSC, and NPC datasets, supporting its potential prognostic value. While our findings integrate transcriptomic meta-analysis, single-cell resolution mapping, and exploratory survival analyses, they remain limited by the reliance on mRNA expression without direct proteomic validation, the lack of corroborative in vitro assays, and the probabilistic nature of inferred cell–cell communication results. Therefore, our conclusions should be interpreted cautiously and validated through future experiments, such as CRISPR-based gene editing or proteasomal inhibition assays, ideally in NPC-specific in vitro models, including organotypic co-cultures of tumor and immune cells. Nonetheless, the data support the hypothesis that targeting UPS activity and restoring antigen presentation may enhance immune recognition, while simultaneously disrupting pro-survival and stemness pathways, offering multiple therapeutic avenues for EBV-associated NPC.

## Methods

### RNA-Seq and microarray datasets

Publicly available RNA-Seq and microarray datasets were obtained from the GEO database (https://www.ncbi.nlm.nih.gov/geo) using the following accession numbers: GSE68799, GSE118719^102^, GSE34573^103^, GSE13597^104^, GSE12452^105^, and GSE64634^106^ (Table 1). Each dataset was individually analyzed for differentially expressed genes (DEGs) using the limma (RRID: SCR_010943) R package^10^, comparing normal vs. cancerous tissue. P-values were adjusted using the false discovery rate (FDR) correction method to account for multiple hypothesis tests. Significant DEGs from RNA-Seq and microarray experiments were subsequently grouped for downstream meta-analysis.

**Table 1:** Publicly available transcriptomics datasets used in the meta-analysis.

GEO Accession Codes	Experiment Type	Experimental Design
GSE68799	RNA-Seq	42 nasopharyngeal carcinomas; 4 normal mucosae
GSE118719	RNA-Seq	7 nasopharyngeal carcinomas; 4 normal mucosae
GSE34573	Microarray	16 nasopharyngeal carcinomas; 4 normal controls
GSE13597	Microarray	25 nasopharyngeal carcinomas; 3 normal controls
GSE12452	Microarray	31 nasopharyngeal carcinomas; 10 normal controls
GSE64634	Microarray	12 nasopharyngeal carcinomas; 10 normal controls

### Meta-regression analysis of DEGs

To account for the heterogeneity of datasets from RNA-Seq and microarray procedures, a meta-regression was conducted on the DEGs using the logFC as the measure of effect size and the standard error as the measure of variance. The standard errors were Winsorized at the 5th and 95th percentiles to minimize extreme values. The meta-regression was conducted using a random-effects model with the restricted maximum likelihood (REML) estimation method, incorporating platform type as a moderator to account for batch effects. For each gene, the following model was applied:

$$\:{logFC}_{i}=\:{\beta\:}_{0}+{\beta\:}_{1}\:\times\:{Platform}_{i}+\:{\epsilon\:}_{i}$$

β_0_ represents the estimated overall effect size, β_1_ represents the platform-specific variations, and ε_i_ represents the residual variance of platform *i*. The model was implemented with robust variance estimation to handle within-study correlations. For genes where the model failed to converge, effect sizes and p-values were set to NA. Using the Benjamini-Hochberg method, a false discovery rate correction was applied to the p-values. All analyses were conducted in R using the metafor package. (Viechtbauer, 2010).

### EBV virus-host protein-protein interaction network analysis

Virus-host protein-protein interaction (PPI) data were obtained from the VirHostNet (RRID: SCR_005978) database (https://virhostnet.prabi.fr/) to identify host proteins interacting with EBV proteins. The PPI dataset overlapped with the DEGs previously identified from the meta-regression analysis. The resulting overlaps were then visualized using Cytoscape (RRID: SCR_003032)^109^ and processed using the Molecular Complex Detection (MCODE) algorithm (RRID: SCR_015828)^110^ to identify the highest interconnected clusters. This approach enables the detection of relevant gene clusters that may not be readily observed. Identified clusters were enriched using the Reactome database (Milacic et al., 2024) to elucidate key biological processes and pathways associated with EBV-host interactions.

### Single-Cell RNA sequencing analysis

Single-cell RNA sequencing (scRNA-seq) datasets were obtained from the GEO database using the following accession numbers: GSE150825^112^, GSE162025^113^, GSE150430^114^, GSE226620^115^, and GSE182227^116^. The datasets GSE150825, GSE162025, and GSE150430 represent nasopharyngeal carcinoma (NPC), while GSE226620 and GSE182227 represent oropharyngeal carcinoma (OPC). OPC datasets were included as EBV-negative cancer controls due to their similar anatomical location and comparable typical histological characteristics to NPC. Enabling direct comparison between the gene expression of EBV-associated NPC and EBV-negative head and neck cancers. The OPC dataset was included solely as an anatomical comparator and was removed for all subsequent analysis after comparative gene expression analysis. Each dataset was processed using Seurat (RRID: SCR_016341)^117^ in R through normalization, scaling, and dimensional reduction measures such as principal component analysis (PCA) and Uniform Manifold Approximation and Projection (UMAP). The dataset GSE150430 was pre-normalized and thus directly used in downstream analysis. The datasets were integrated using the Harmony algorithm^118^ to correct batch effects and differences in pathological conditions. Low-quality cells were removed using the following thresholds: cells with < 200 detected genes, genes detected in fewer than 3 cells, or cells with excessive mitochondrial RNA content (thresholds were determined specific to each dataset). Counts were log-normalized using log normalization (factor = 10,000), and the top 20 highly variable genes were identified and used as anchors. The GSE150430 dataset was incorporated directly into downstream integration steps. The data were then scaled, and PCA was performed to obtain a low-dimensional representation. The top 30 principal components were then used as input for integration with the Harmony algorithm, using the GEO sample ID as the batch covariate. The default parameters for Harmony integration were used. Batch correction was visually assessed by generating UMAP embeddings both before and after Harmony integration of Harmony. After integration, the UMAP plots showed that cells clustered by biological identity rather than dataset origin.

Two of the three single-cell datasets (GSE162025 and GSE150430) contained EBV transcript information, which we used to verify the clusters for evidence of EBV infections (Supplementary Materials 7). After integration, the cells were clustered using the FindClusters function, and marker genes were identified using FindAllMarkers using Seurat. The top five marker genes were selected for further enrichment to identify cluster identities (Supplementary Materials 3). The enrichR (RRID: SCR_001575)^119^ package was used to enrich the top marker genes from each cluster against multiple reference databases. Two independent authors verified the cluster identities to ensure accuracy.

### Cancer cells classification using InferCNV and EBV transcript positivity

The InferCNV (RRID: SCR_021140) R package^120^ assessed large-scale chromosomal alterations in single cells. This analysis aimed to identify genomic instability in the cell clusters. The following immune and stromal cells were used as reference groups: endothelial, dendritic, and regulatory T cells. The cells with heavy genomic instability, as shown by increased or decreased predicted copy number variations, were then classified as cancer cells. These cells were further examined for EBV transcript positivity. However, only two datasets contained information for the viral transcriptomes. Therefore, the annotations were incomplete. Additionally, the low read counts also meant that we might not find lowly expressed EBV transcripts. Nevertheless, clusters that expressed these transcripts were deemed to be cancer cells (Supplementary Materials 7). Finally, epithelial lineage markers (EPCAM, KRT8, KRT18, CDH1) were used to confirm epithelial identity. Clusters lacking these features were excluded from downstream cancer-cell analyses.

### Immune pathway correlation analysis

To evaluate pathway activity at the single-cell level, scoring was performed using the AUCell (RRID: SCR_021327) package (v1.20.1) with gene sets from the Gene Ontology Biological Process from the MSigDB (RRID: SCR_016863) database. Rankings of gene expression were computed using AUCell. The top 100 most variable gene sets were selected, and their AUC scores were correlated with the average expression of the UPS-associated gene signature (*CCT6A*,* CCT3*,* PSMA1*,* PSMC4*,* PSMA7*,* PSMA6*,* PSMA4*,* PSMD1*,* PSMD14*,* PSMD12*,* UCHL5*,* and CCT5*) using Spearman’s correlation. For each gene set, both the correlation coefficient (ρ) and the corresponding p-value were computed, and the p-values were adjusted using the Benjamini-Hochberg method. To highlight immunologically relevant biological processes, GO terms containing immune-related keywords (e.g., “IMMUNE”, “T CELL”, “CYTOKINE”, etc.) were filtered from the correlation results. The top 10 positively and top 10 negatively correlated immune-related gene sets were visualized.

### Cancer cell reclustering and classification

Cells were reclustered to investigate the role of proteasomal function in nasopharyngeal carcinoma. We conducted sensitivity analysis of multiple resolution parameters (0.2–1.0) in Seurat and compared them using a heatmap of the top 5 marker genes per cluster (Supplementary Materials 4). We selected 0.2 for the final resolution due to the optimal separation of the markers while maintaining that several clusters are transcriptionally distinct. For each cluster, the expression of UPS-associated genes was measured. Specifically, the average expression of *CCT6A*,* CCT3*,* PSMA1*,* PSMC4*,* PSMA7*,* PSMA6*,* PSMA4*,* PSMD1*,* PSMD14*,* PSMD12*,* UCHL5*, and *CCT5* was calculated, and the cells were classified into UPS-high and UPS-low groups based on the expression of these genes. The cell groups were further assessed for cell proliferation by calculating the cell cycle states in Seurat^117^. The proportion of cells in G1, G2/M, and S states was compared between the two groups using Pearson’s Chi-Square test.

### Cell-cell communication analysis using cellchat

Cell-cell communication analysis was performed using the CellChat (RRID: SCR_021946) package in R^121^. NPC datasets were divided into two groups (UPS-high and UPS-low) to explore alterations in intercellular signaling, and each network was inferred separately. The ligand-receptor interactions were ranked based on interaction weight, and the patterns were compared between the two groups. Differential expression analysis was performed on ligand-receptor pairs with UPS-low cells designated as the reference group. Upregulated and downregulated signaling interactions were identified and mapped to ligand-receptor pairs. Bubble plots were used to visualize significantly upregulated and downregulated ligand-receptor interactions in UPS-high cancer cells. Network ranking plots were also created to compare the input and output signaling from the UPS-high and UPS-low cancer cells.

### Survival analysis of the UPS genes using GEPIA2

Survival analysis was performed using the GEPIA2 (RRID: SCR_026154) database (http://gepia2.cancer-pku.cn)^122^ to evaluate the prognostic relevance of the 12 genes associated with UPS (*CCT6A*,* CCT3*,* PSMA1*,* PSMC4*,* PSMA7*,* PSMA6*,* PSMA4*,* PSMD1*,* PSMD14*,* PSMD12*,* UCHL5*, and *CCT5*). Each gene was individually assessed for its associations with overall survival across TCGA cancer types using the Survival Map module. Genes with log-rank test *p* < 0.05 were considered statistically significant. To assess the combined effect of the 12-gene set, Kaplan–Meier survival curves were generated using the Survival Analysis module for two datasets: the full TCGA pan-cancer data, encompassing all available tumor types, the TCGA head and neck squamous cell carcinoma (HNSC) data, and a smaller NPC-specific dataset. We conducted multiple analyses due to the relatively small sample size of the NPC datasets. Patients were stratified into high- and low-expression groups based on median expression of the combined signature.

Additionally, a separate survival analysis was also conducted using an NPC transcriptomic dataset containing disease progression information to explore the potential clinical relevance of the UPS signature within EBV-associated NPC. RNA-seq count data (GSE102349)^30^ were processed in R^123^ using the DESeq2^124^ package. The 12-gene ubiquitin–proteasome system was computed as the average of z-scored expression values for these genes in each sample. Patients were classified into high-UPS and low-UPS groups using the median value as a cutoff. Survival analyses were performed using the survival and survminer packages, with progression-free survival as the primary endpoint^125^. After removing incomplete data, 88 patients remained for analysis. Kaplan–Meier curves were generated, and the log-rank test assessed differences. Hazard ratios (HR) with 95% confidence intervals were estimated by Cox proportional-hazards regression.

## Supplementary Information

Below is the link to the electronic supplementary material.

Supplementary Material 1

Supplementary Material 2

Supplementary Material 3

Supplementary Material 4

Supplementary Material 5

Supplementary Material 6

Supplementary Material 7

## Data Availability

The public dataset used in the analysis are available from the GEO database from the following accession codes GSE68799, GSE118719, GSE34573, GSE13597, GSE12452, and GSE64634 for bulk transcriptomics data and GSE150825, GSE162025, GSE150430, GSE226620, and GSE182227 for the single-cell RNA Sequencing data. The code and script used in the analysis are available on figshare with doi:10.6084/m9.figshare.29900015.
