# Supplementary material for: The ubiquitin–proteasome system is an important driver of EBV-associated nasopharyngeal carcinoma progression: a meta-analysis of transcriptomic data
Source: Sci Rep. 2026 Feb 24;16:8892. doi: 10.1038/s41598-025-34808-4 (PMC12987939; doi:10.1038/s41598-025-34808-4)
Supplement: Supplementary file 4 — Supplementary Material 4 [file 41598_2025_34808_MOESM4_ESM.docx]

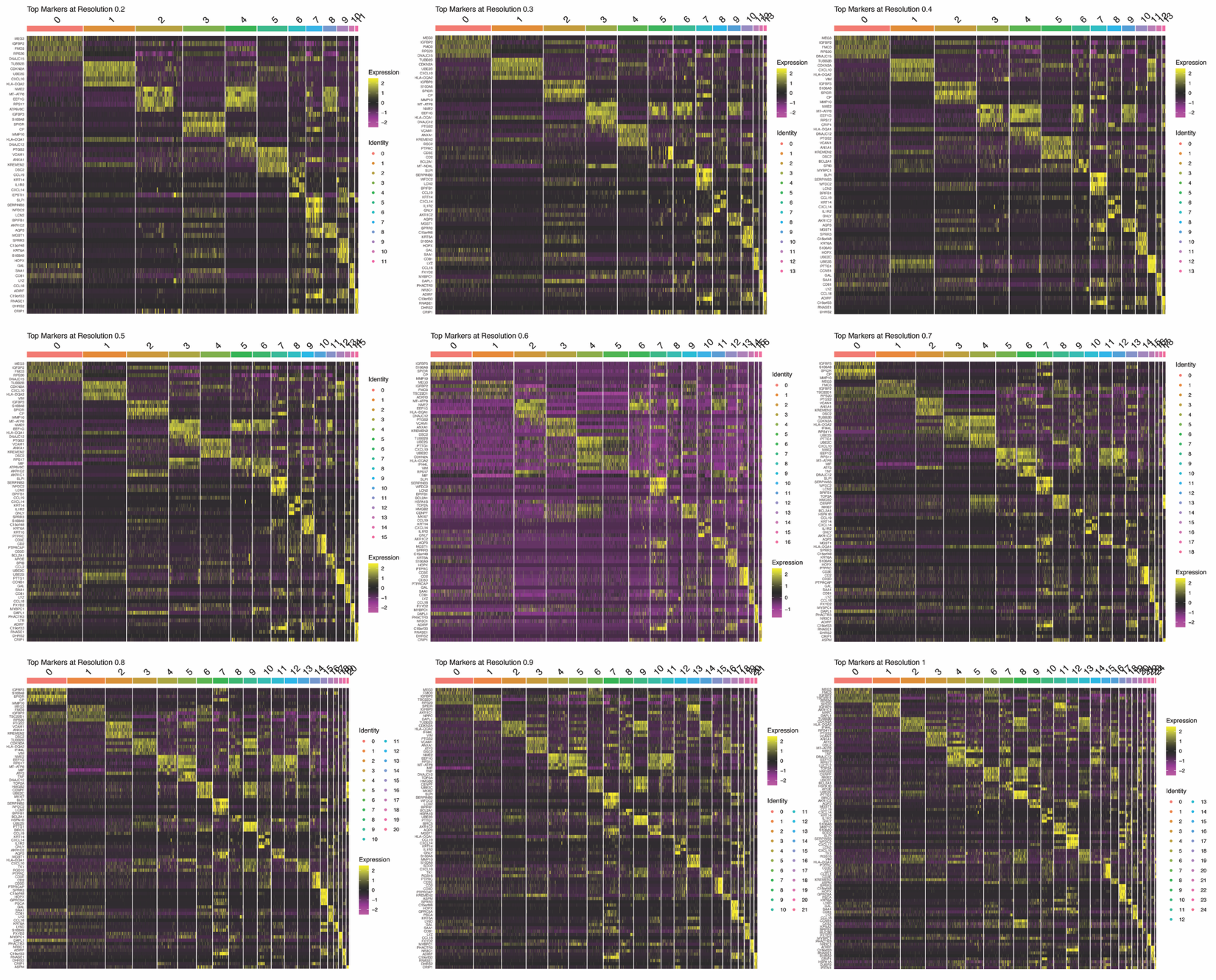


**Supplementary Materials 4. Heatmaps of the top marker genes across multiple clustering resolutions (0.2–1.0) of cancer cells**. Resolution 0.2 yielded the fewest clusters while maintaining clear separation between groups and minimal marker overlap, representing the most interpretable configuration.
