# Supplementary material for: The ubiquitin–proteasome system is an important driver of EBV-associated nasopharyngeal carcinoma progression: a meta-analysis of transcriptomic data
Source: Sci Rep. 2026 Feb 24;16:8892. doi: 10.1038/s41598-025-34808-4 (PMC12987939; doi:10.1038/s41598-025-34808-4)
Supplement: Supplementary file 7 — Supplementary Material 7 [file 41598_2025_34808_MOESM7_ESM.docx]

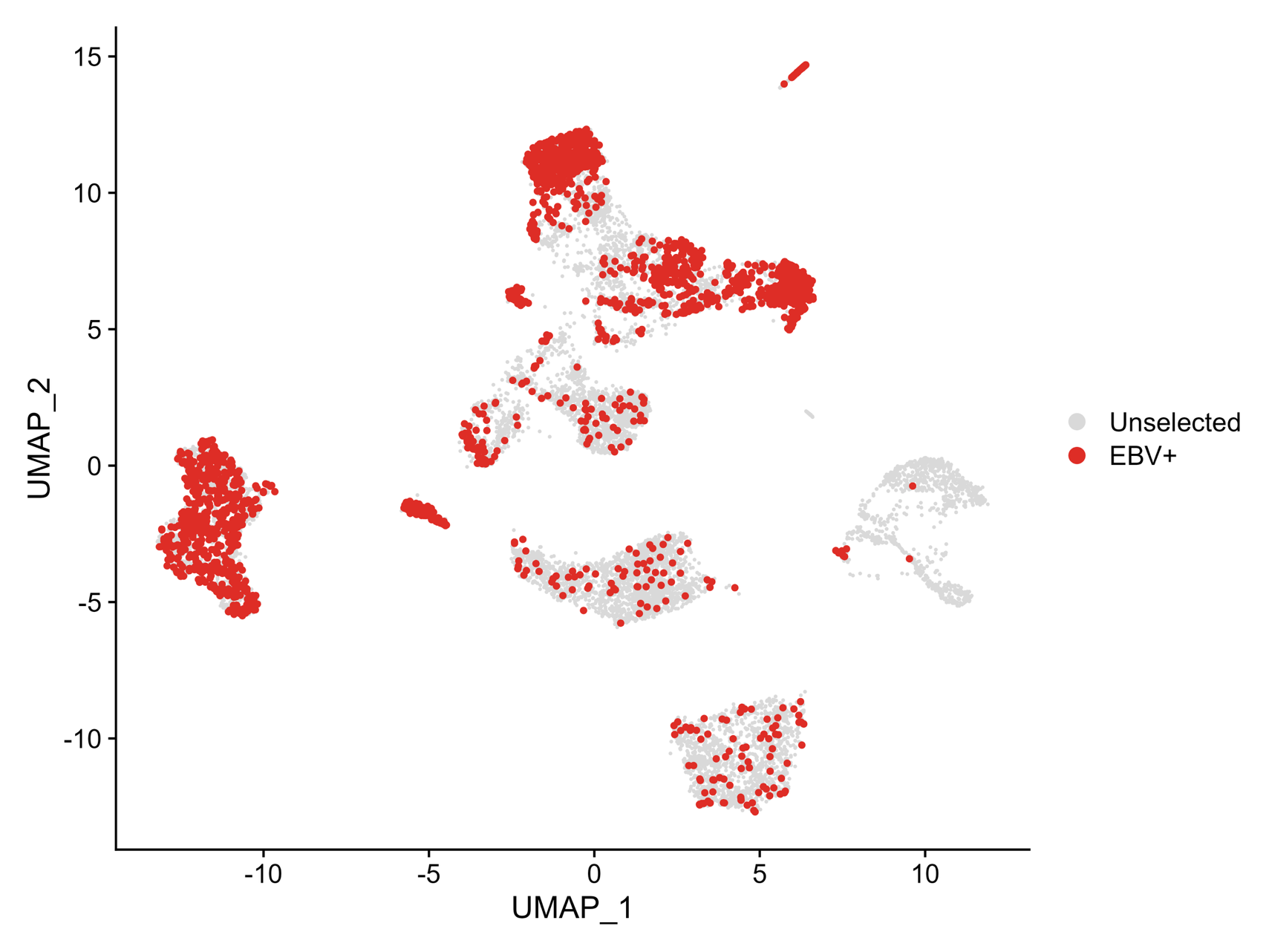


**Supplementary Materials 7. Mapping of cells expressing at least one EBV transcript across NPC clusters.** Cells expressing EBV transcripts are shown in red, while EBV-negative cells are shown in gray. EBV transcript mapping was available for only two of the three NPC datasets, and the relatively low read depth likely limited detection sensitivity, causing some clusters to appear sparsely labeled.
